# Analysis of fit on implants of chrome cobalt versus titanium frameworks made by cad / cam milling

**DOI:** 10.4317/jced.57817

**Published:** 2020-10-01

**Authors:** Begoña Oteiza-Galdón, Amparo Martínez-González, Ángel-Vicente Escuder

**Affiliations:** 1 Faculty of Dentistry, European University of Valencia, Spain; 2 Polytechnic University of Valencia

## Abstract

**Background:**

This study analyzed the degree of passive and vertical fit achieved in frameworks using either cobalt-chromium (Co-Cr) or titanium (Ti) implant-supported fixed partial dentures ( FPDs) fabricated with a CAD/CAM milling technique.

**Material and Methods:**

33 3-unit FDPs, 17 of Co-Cr metal alloy (test group) and 16 of Ti (control group), were manufactured with two implants by copy milled technology. Optical microscopy was used to measure passive fit (PF) and vertical fit (VF) in all frameworks. The PF was evaluated by using the Single Screw test and the VF with the screws tightened at 20 Ncm. Descriptive and inferential analysis were performed to evaluate statistically significant differences in the tested groups for each fit. Brunner-Langer models were applied to assess potential material and implant area effects on the measurements. An ANOVA test was performed to estimate both main effects and interactions.

**Results:**

The average PF values in the screwed implant were 4.43 ± 0.52 µm for Ti and 5.50 ± 1.61 µm for Co-Cr and in the non-screwed implant 5.59 ± 1.32 µm in the group Ti and 6.25 ± 1.55 µm the Co-Cr group. In this last implant, it was not observed statistically significant differences between both types of alloy (*p* = 0.178) nor between zones. Ti control group exhibited a significantly better VF than Co-Cr (*p* = 0.046) in the screwed implant but there were no differences in the implant not screwed. The VF in the non-screwed implant was better in lingual than in buccal zone.

**Conclusions:**

The PF and VF measurements observed in Co-Cr frameworks are clinically acceptable. 3-unit implant supported FPDs made with Co-Cr alloy using milling technique showed similar dimensional accuracy than those obtained with Ti.

** Key words:**Cobalt- chromium alloy, misfit, passive fit, vertical fit, milling.

## Introduction

A major clinician concern when placing prostheses on implants is achieving a good suprastructure fit. It is considered that a structure has passive fit when there is a continuous contact between the implant and prosthesis connection surfaces (1-3) not generating tension or stress before loads application (4,5). The discussion on the accuracy of the fit is based on the biomechanical fact that the tightening of the screws to seat the prosthesis on the implants introduces stress in the implant-prosthesis interface that is then transmitted to the bone and to the different components of the implant-abutment complex. Prosthesis misfit is associated with biological complications, such as bacterial microleakage in the microgaps at the interface and peri-implant bone loss, and mechanical complications including untightening or breakage of the retaining screws, prosthesis fractures, and even implant fracture (5,6).

 The prosthesis material and the manufacture technique can influence the degree of misfit in frameworks. The conventional casting method in prosthesis fabrication involves a series of steps with considerable human intervention and manipulation of materials that, inherent exhibit contractions and / or expansions, which may cause processing errors and inaccuracies (7,8).

 Although material casting studies generally show better adjustment of suprastructures on implants using noble alloys rather than alloys of base metals (9-12), their high cost implies limitations that leads to the development of new alloys. Co-Cr alloys offer important advantages over noble alloys, including their lower cost and better physical properties, despite exhibiting greater corrosion than other metals. Although Ti is very resistant to corrosion, due to its physical characteristics, Ti casting is a highly complex method requiring special apparatus and advanced technology, which leads to lower fit accuracy than the one achieved with noble alloys.

CAD / CAM design and manufacturing has transformed industry in general and particularly in the dentistry field. This technology significantly increases structural accuracy by reducing the sources of error. Moreover, being a deeply automated process, it dramatically shortens manufacturing time, efficiently reduces costs and lessens dependence on highly skilled and experienced laboratory technicians (13).

Within the past few years, the application of this advanced technology to perform implant prostheses of Titanium is consistently achieving an optimum degree of fit (14-17). Being now available the use of CAD / CAM-manufactured Co-Cr structures to perform fixed implant-supported prostheses seems an ideal opportunity: Co-Cr CAD / CAM improves the fit with respect to Co-Cr casting; moreover, it also reduces costs caused by the use of UCLA abutments with mechanized base for overcasting with noble alloys (11,12); in addition, Co-Cr-based suprastructures assures optimal adhesive bond to ceramics due to its ability to join them through the formation of oxides (18), which also reduces complications caused by ceramic fractures. The purpose of this study was to evaluate the degree of passive and vertical fit achieved with CAD/CAM-manufactured Co-Cr implant frameworks, compared to those achieved with Ti implant frameworks built through a similar protocol.

## Material and Methods

-Framework fabrication

The research sample consisted of 33 fixed 3-unit implant-supported fixed partial dentures ( FPDs) manufactured by CAD/CAM milling technology using either a Co-28Cr-6Mo cobalt-chromium metal alloy ( n=17) and a Ti-6Al-4V Extra Low Interstitial (ELI) titanium alloy (n=16). 66 Biomet 3i conical internal connection implants (Biomet3i, Palm Beach Gardens, FL, USA) were used. Two implants were placed on a cylindrical resin base with a separation of 10 mm. To maintain the position of the implants, a plastic piece was manufactured. Epoxy resin (EpoFix, Struers, Cleveland, OH, USA) was then prepared and poured onto the cylinder so that the implants were submerged in the resin with the implant connection platform 3 mm above it to facilitate both their design and scanning processes. The implants were designated A and B. The implant A was placed in the mandibular left first premolar position and the implant B in the first molar position. 

Once the first specimen was manufactured, in order to obtain the rest of the samples in the same implant position, two impression copings were screwed on the implants and a silicone key was made in a way that it embedded the impression copings. To obtain the rest of the specimens, implants were screwed to the impression copings and covered by the epoxy resin inside the cylindrical base. In this way all specimens were manufactured.

**Figure 1:**
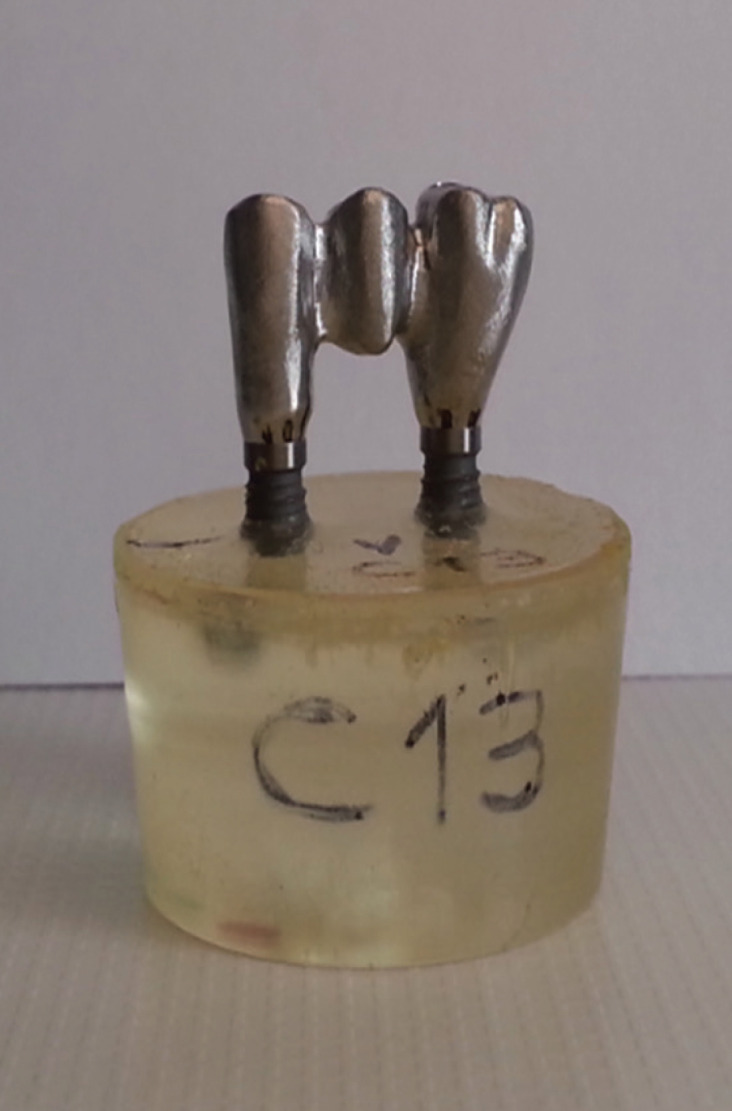
Research specimen.

On the first sample, a waxing of the structure with the final anatomical shape was made on temporary abutments. Thus each experimental specimen and the waxed framework were scanned. From these scans the frameworks of the tested alloys were manufactured at the Biomet 3i Milling Center by copy milling a block of correspondent material (Fig. 1).

-PF and VF Measurement

For all superstructures, passive fit (PF) and marginal vertical fit (VF) were analyzed by optical microscopy (OM) with a Leyca OM (Leyca Microsystems Ltd., Wetzlar, Germany) and an external light source.

**Figure 2:**
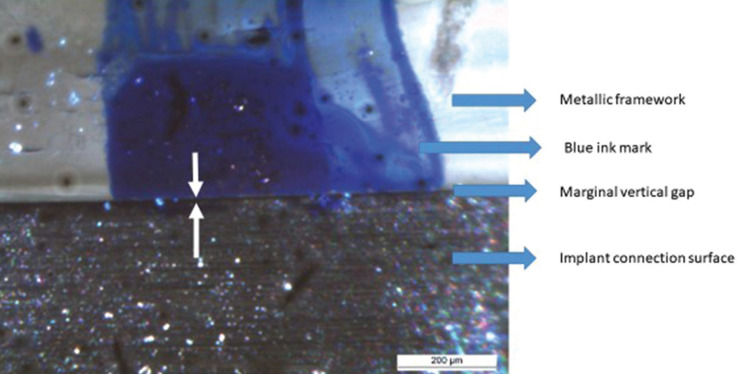
OM Image of the implant-abutment interface.

PF was measured according to the single screw test also known as The Sheffield test, tightening first one screw at one end of the framework, implant A, to observe then the discrepancies at the other implant abutment, implant B. Thus, for each specimen, the implant A screw was manually tightened up to a roughly 10 Ncm torque, while leaving the implant B screw untightened. Images of each implant-prostheses interface were obtained at the level of the previously obtained marks (Fig. 2), 2 of the buccal area (Bu) and 2 of the lingual area (L), for a total of 4 images per implant (8 per specimen). On each image, the average of three measurements was obtained.

 For VF analysis, framework screws were placed on implants A and B with a torque of 20 Ncm, as recommended by the manufacturer, using a manual torque meter (Biomet 3i). Then, a set of 8 images per specimen were obtained and their 8 correspondent measurements (averages of triplicates) were obtained as previously described for PF analysis.

-Statistical analysis 

Descriptive statistics (mean, standard deviation, range and median) were calculated for the variables being analyzed in both groups (control/test) and inferential analysis was performed to evaluate significant differences in those fitting values between groups (Ti and Co-Cr). Due to the sample size in these groups, this analysis was carried out from a non-parametric approach: Brunner-Langer models to assess the effect of the group (control / test) and the area (Bu / L) on the of fitting measurements. In addition, main effects and interactions were estimated from an ATS test (ANOVA type). The significance level applied in the analysis was 5% (α = 0.05).

## Results

-Results of the study of the PF of the frameworks 

The PF for implant A in zone Bu averaged 4.38 ± 0.88 μm in the Ti samples and 5.79 ± 2.26 μm in the Co-Cr samples. In zone L, the fit was also descriptively better in the control group, 4.48 ± 1.07 μm versus 5.21 ± 1.42 μm for Co-Cr. The degree of misfit observed in the screwed A implant is significantly smaller in Ti samples than in Co-Cr for both zone Bu and zone L (*p* = 0.025 and *p* = 0.846, respectively). In non-screwed implant B, where the PF of the suprastructure was really studied, measurements in zone Bu averaged 5.41 ± 1.23 μm in the Ti samples and 6.17 ± 1.75 μm in the Co-Cr samples. In zone L, the adjustment was also descriptively better in the Ti control group than in the experimental group (5.78 ± 1.76 μm and 6.32 ± 1.69 μm, respectively). No statistically significant differences were observed between both types of alloys (*p* = 0.178) or between the two measured zones (*p* = 0.636).

The fitting values of both zones, Bu and L, were averaged. Table 1 describe an average fit in implant A and in implant B showing better results in screwed implant A. In implant B, where the structure was not screwed, the fit was greater in Ti than in Co-Cr frameworks. The Brunner-Langer model emphasizes the significant differences between implants A and B (*p* <0.001): the fit is significantly better in implant A (Table 2). There are no differences between the different alloys; but there is a strong trend (*p* = 0.068), which is consistent with the fact that for implant A it was (*p* = 0.025) and not for implant B (*p* = 0.178), showing a greater fit in Ti frameworks. Regardless of the observed differences or trends by alloy group, all measurements are consistently below 10 μm, as observed in Table included in this study.

**Table 1:**
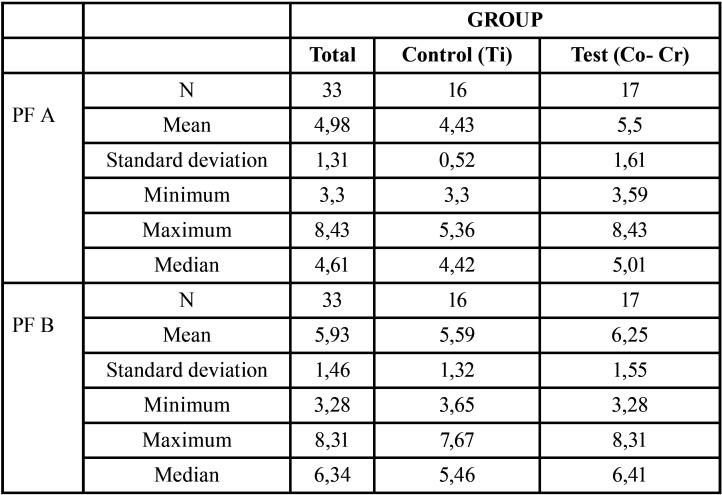
PF of implants A and B (in μm) for each group.

**Table 2:**
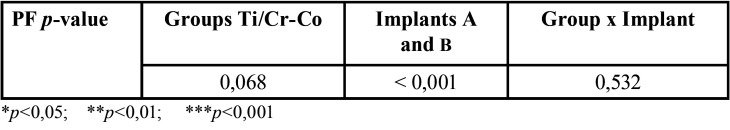
Brunner-Langer models for assessment of passive fit according to group and implant: ATS test.

-Results of the study of the marginal VF of the samples 

VF for implant A in zone Bu averaged 3.99 ± 0.65 μm in the Ti samples and 5.20 ± 1.47 μm in the Co-Cr ones. In zone L, the fit was also better in the Ti control group (4.28 ± 0.87 μm) compared to that in the Co-Cr group (4.99 ± 1.02 μm). The VF measure in implant A was significantly smaller in Ti samples than in Co-Cr (*p* = 0.002). This result was valid for both zones Bu and zone L (*p* = 0.326).

For implant B in zone Bu, Ti samples and Co-Cr samples averaged 4.71 ± 1.51 and 5.19 ± 1.61 μm, respectively. In zone L, the differences in average were barely estimated at 0.5 μm (4.12 ± 0.86 μm and 4.66 ± 1.2 μm for Ti and Co-Cr, respectively). The VF measure in implant B was similar between both groups (*p* = 0.206). However, the fit in zone L showed to be significantly better (*p* = 0.022) than in zone Bu. This result is extrapolated to any type of alloy (*p* = 0.615).

For both implants, we obtained average fit values for both zones Bu and L, as observed in Table 3. The Ti control group exhibited a significantly better fit than the Co-Cr one (*p* = 0.046). Clearly, differences were more significant in implant A than in implant B (Table 4).

**Table 3:**
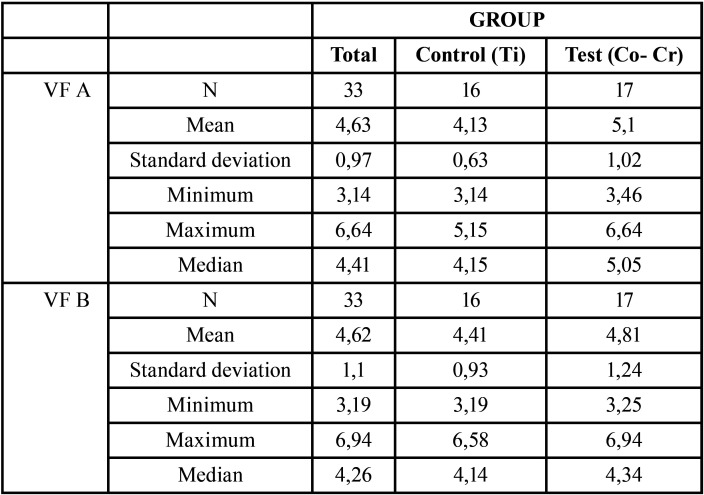
VF in implants A and B for each group (microns).

**Table 4:**
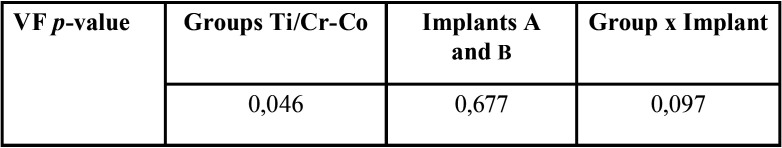
Brunner-Langer models for assessment of vertical fit according to group and implant: ATS test.

## Discussion

The long-term success of implant restorations is related to the ability of the implant-prosthesis system to withstand occlusal forces without generating stress or tension in the peri-implant bone, avoiding screw and suprastructure failures. An aspect related to this success depends on the passivity of the structure on the implants (19). This *in vitro* study compares the fitting accuracy of 3-unit screw-retained FDPs supported on 2 implants, manufactured with either an alloy of Co-Cr or Ti by milling technology. All built structures had a certain degree of misfit, so there is no passive fit in absolute terms.

The passive misfit values obtained are small, lower than 11 μm in both types of implants, similar to those reported in a study by Svanborg *et al.* (17). In addition, the quality of the fit is similar in all the coupling areas of the structure, both in the buccal and lingual areas analyzed, indicating that the fit is homogeneous. Independently, of the material used, the fit is greater in the implant with the tightened fixing screw than in the unscrewed implant, which is expected since, although manually, a tightening torque has been applied. Regarding the materials, although the fit is significantly better for the Ti both in the tightened and the non-tightened implants, being the latter where a PF was really assessed, we did not find significant differences, reflecting a similar PF for both materials.

The fit values obtained in this study are lower compared to those reported in other studies (20,14-16). First, differences may derive from using a different measurement method, MO and SEM, and additional differential details in the methodology of the studies. In fact, variations in the fitting values can be expected when using different measurement methods for a given structure. Furthermore, some differences in the fitting values of the structures may be related to the control and management of the manufacturing technique. The better the control of the entire manufacturing process, the better the adjustment results.

According to this study, the results of PF in the non-screwed implants were better with the specimens of milled Ti than in those of Co-Cr, 5.59 ± 1.32 μm and 6.25 ± 1.55 μm, respectively, although there were no significant differences. This misfit can be a consequence of the distortion that milling introduces to the metal structure. Milling is a hard or post-machining process in which the exact dimensions of the restoration are milled. The technique involves high cutting speeds under pressure, generating high temperatures in the material block. Due to the hardness of the material, intense cutting forces are required to achieve efficient removal of the material, which generates greater heat in materials with low thermal conductivity such as Ti. Constant cooling, using a mixture of water and oil in this case, is 

required to prevent overheating. These milling conditions generate both mechanical and thermal stress that cause a distortion in the outer layer of the structure (21) that seems to be similar for both materials.

From these results we can deduce that the milling technique generates similar surfaces in terms of 

roughness and degree of distortion for Ti and Co-Cr, which allows obtaining similar passive fit values. In addition, the misfit values obtained are below the physiological limit set at about 150 μm. These values are within the level of bone tolerance to the lack of adaptation of the prosthesis to the implants and are therefore considered clinically acceptable. This allows us to affirm that with the CAD / CAM technique we can achieve Co-Cr structures of great quality and accuracy in terms of adjustment. As a clinical consequence, the choice of material for milling CAD / CAM seems to have a minor effect on the passivity of the adjustment, while the accuracy of the master model seems to be decisive. In this sense, conventional impression scanning technology or intraoral scanning is important.

When the marginal VF was analyzed, all the screws were tightened to the torque indicated by the manufacturer, 20 N, applied with a calibrated torque meter. When this happens, the gap size decreases for both materials obtaining better vertical fit values than those obtained in the initial passive fit, which occurs in both implants, being more apparent in the implant not initially screwed. Our results are consistent with those presented by De França *et al.* (14) and by Abduo *et al.* (21), who also observed a significant reduction in the gap.

When assessing the passive adjustment in the non-screwed implant, the implant-prosthesis contact is initially limited to the irregularities that exist on their surfaces, but those irregularities flatten at a later stage by applying the tightening torque. Such surface fixation occurs when a force is exerted throughout the metal interface and depends on the initial surface roughness, hardness, and the magnitude of the torque of tightening and loading (22). In addition, greater fixation can be expected with internal connection implants relative to that using external connection implants (22). This mechanism would explain how applying torque improves the fit of both implants with respect to passive fitting values in our research.

On the other hand, extrapolating this effect to the mouth of the patients, a flexion of the suprastructure and bone also occurs upon tightening of the screws (23). To simulate this effect, we used an epoxy resin with an elastic modulus similar to that of cortical bone (15.0 GPa), which makes it representative of a natural jaw. It would be considered equivalent to a type I bone quality of the 

Lekholm and Zarb classification (24). The suprastructure moves vertically towards the implant in combination with a rotational movement in synchrony with the vertical movement of the implant head towards the suprastructure. When the passive vertical misfit increases, the suprastructure is subjected to greater deformation to close the vertical gap at the time the screws are tightened (23). In this way, misfits of 50 to 100 μm can be closed in clinical situations. This flexion generates tension in the implant-prosthesis connection and in the surrounding bone due to the natural ankylosis caused by bone integration (19). Thus, as the magnitude of the misfit increases, tightening the screws reduces the gap but gradually increases the tension, giving a false sense of adjustment. Thus, there is always a residual static stress related to the gap size.

Regarding the material, Titanium structures showed a better fit than Co-Cr after tightening all retaining screws. In implant A, the fit was significantly better with Ti than with Co-Cr; In implant B, the fit was similar with both materials and, notably better in the lingual area. Comparing the mean values of the two materials, the fit improves with Ti, consistent with its lesser stiffness and, therefore, a tendency to suffer greater deformation when tightening the screw and closing the gap.

All the measured values obtained for both materials were below 10 μm. Previous studies comparing different construction materials for CAD / CAM structures, such as Zir and Ti (21) or Zir and Co-Cr (14), obtained similar vertical fit values for all materials. In any case, the fitting differences observed in Co-Cr and Ti may have minimal clinical relevance. In patients, we can expect greater misfit than that observed *in vitro* as additional misfit might be introduced at different levels, including the elaboration of the impressions necessary to obtain the working model for the structure and the introduction of additional variables not considered in this study, as a greater number of implants or their disparallel position. Therefore, when constructing structures using this technology, the misfit derived from the manufacturing technique seems more relevant that the selection of material and valid vertical fitting values can be obtained using either Co-Cr alloys or titanium.

Previous studies analyzing stress transmission upon screws tightening found no differences between the materials (25,15). In our study, with values of vertical adjustment for the Co-Cr alloy below 10 μm and with a high elastic modulus, higher than that of Ti, load application would be expected to concentrate stress in the structure rather than in the bone. At the same time, the inherent rigidity of the structure would provide greater protection of the retaining screws and also less tension in the ceramic coating. Consequently, there would be fewer mechanical complications associated to the prosthesis.

 Previous clinical studies have not concluded that (more rigid) Co-Cr suprastructures are prone to fail. An 18-year retrospective analysis by Teigen *et al.* (26) aimed at verifying the clinical results of cast screwed suprastructures made of either type III gold or Co-Cr coated with either ceramic or resin found similar clinical results for the two types of materials. Moreover, the study showed that the 18-year survival rate and 15-year success rate of the implant was 96% and 90-95%, respectively. Thus, the selection of the biomaterial does not seem to influence the success or survival of the implant (26).

## Conclusions

Both PF and VF measured in the Co-Cr alloy frameworks are clinically acceptable. The fit in Titanium samples is higher than in Cobalt-Chromium samples. The PF and VF reached the statistical significance in the screwed implant, but not in the implant without screwing. There are no differences in PF with respect to the buccal or lingual area of the measurement but the VF was better in the lingual area than in the buccal one. 3-unit implant supported FPDs made with Co-Cr alloy using milling technique showed similar dimensional accuracy than those obtained with Ti.
